# Development of pathway-oriented screening to identify compounds to control 2-methylglyoxal metabolism in tumor cells

**DOI:** 10.1038/s42004-023-00864-y

**Published:** 2023-04-13

**Authors:** Kouichi Yanagi, Toru Komatsu, Yuuta Fujikawa, Hirotatsu Kojima, Takayoshi Okabe, Tetsuo Nagano, Tasuku Ueno, Kenjiro Hanaoka, Yasuteru Urano

**Affiliations:** 1 Graduate School of Pharmaceutical Sciences, The University of Tokyo, 7-3-1 Hongo, Bunkyo-ku, Tokyo, 113-0033 Japan; 2 School of Life Sciences, Tokyo University of Pharmacy and Life Sciences, 1432-1 Horinouchi, Hachioji-shi, Tokyo, 192-0392 Japan; 3 Graduate School of Pharmaceutical Sciences, Keio University, 1-5-30, Shiba-koen, Minato-ku, Tokyo, 105-8512 Japan; 4 Graduate School of Medicine, The University of Tokyo, 7-3-1 Hongo, Bunkyo-ku, Tokyo, 113-0033 Japan; 5 Present Address: Chugai Foundation for Innovative Drug Discover Science, 4-11-5 Nihonbashi Honcho, Chuo-ku, Tokyo, 103-0023 Japan

**Keywords:** Oxidoreductases, Chemical tools, High-throughput screening, Fluorescent probes, Screening

## Abstract

Controlling tumor-specific alterations in metabolic pathways is a useful strategy for treating tumors. The glyoxalase pathway, which metabolizes the toxic electrophile 2-methylglyoxal (MG), is thought to contribute to tumor pathology. We developed a live cell-based high-throughput screening system that monitors the metabolism of MG to generate d-lactate by glyoxalase I and II (GLO1 and GLO2). It utilizes an extracellular coupled assay that uses d-lactate to generate NAD(P)H, which is detected by a selective fluorogenic probe designed to respond exclusively to extracellular NAD(P)H. This metabolic pathway-oriented screening is able to identify compounds that control MG metabolism in live cells, and we have discovered compounds that can directly or indirectly inhibit glyoxalase activities in small cell lung carcinoma cells.

## Abstract (web summary)

The 2-Methylglyoxal (MG) metabolic pathway shows significant correlation with the development of various tumor cells, however, screening pathway inhibitor using live cell is still challenging. Here, the authors design a membrane impermeable probe and develop a live cell-based high-throughput screening system to identify compounds that control and perturb MG metabolism.

## Introduction

**Fig. 1:**
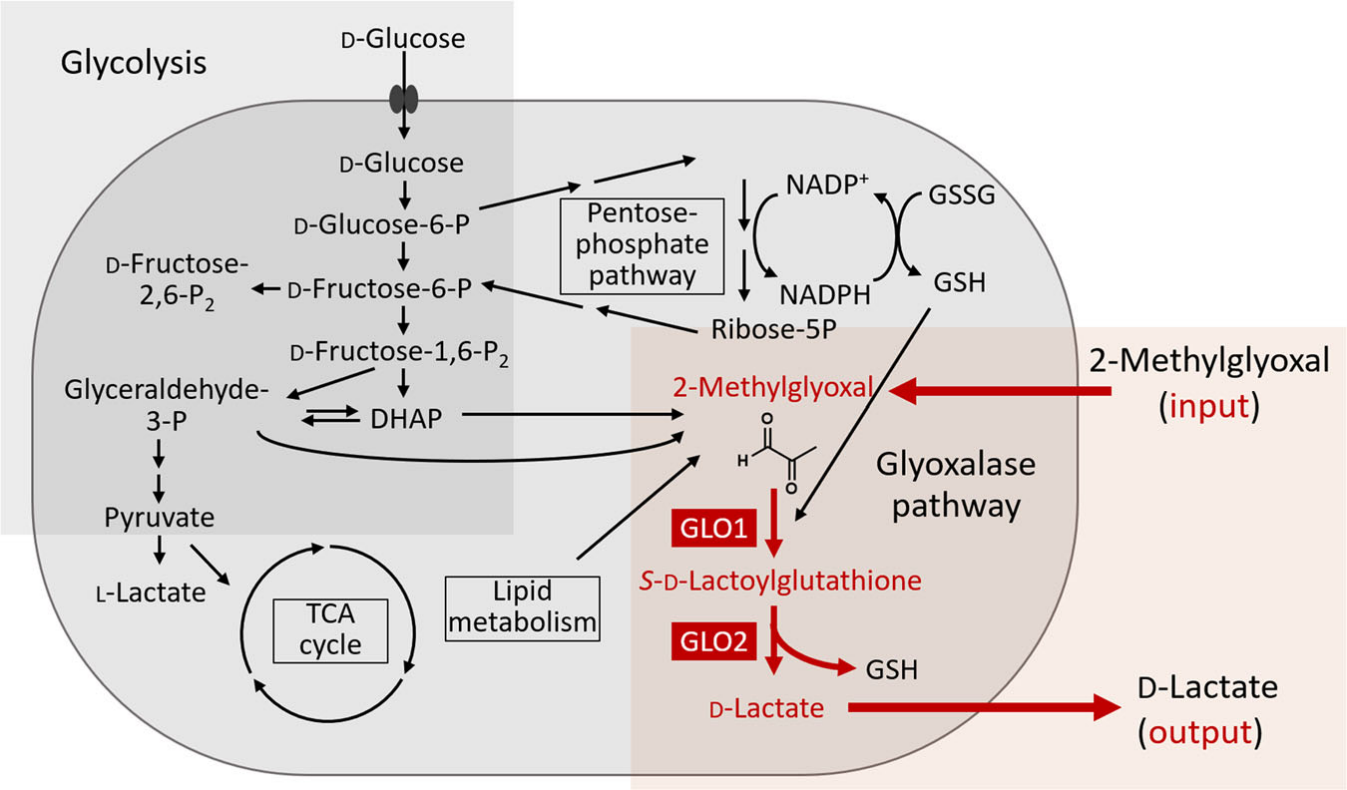
Design of metabolic pathway-oriented screening to control glyoxal metabolic pathway. Targeted pathway connecting the input and output metabolites used for the screening (2-methylglyoxal and d-lactate) is shown in orange rectangle, and the important intermediate metabolites are shown in red letters.

Cellular metabolism plays a central role in homeostasis and its alterations are tightly associated with the development of diseases. Controlling the metabolic pathways is a vital strategy in drug development^1,2^. 2-Methylglyoxal (MG) metabolic pathway is one of the pathways that show significant elevations in various tumor cells and their members, glyoxalase I (GLO1) and glyoxalase II (GLO2), are considered as important drug targets (Fig. 1)^3^. One of the characteristic metabolic changes in tumor cells is the elevation in glycolytic activities (Warburg effect), which results in increased generation of MG as a downstream metabolite^4^. The electrophile reacts with proteins, DNA molecules, and phospholipids to exert toxic effects (dicarbonic stress) in the cells^5^. The MG-metabolizing system counteracts this effect; therefore, increased glycolysis is often accompanied by an increased MG-metabolizing system in tumor cells, and blocking it is known to cause a cytotoxicity by accumulation of MG (Table S1)^6^. MG forms the adduct with cellular glutathione (GSH), and GLO1 catalyses the generation of *S*-lactoylglutathione (SLG) from the adduct, and GLO2 facilitates the metabolism of this intermediate to regenerate GSH and release d-lactate (Fig. 1). Glyoxalase activity is elevated in various tumours, including gastric^7^, breast^8^, liver^9^, pancreatic^10^, lung^6^, and colorectal^11^ tumours. Despite the potential importance of the inhibitors of this pathway as anti-cancer agents, glyoxalase inhibitors have not been fully developed, partly due to a lack of methods to monitor their activities in live cells (Table S2)^5^. High-throughput screening (HTS) using large compound libraries is a powerful strategy for developing new inhibitors, and live cell-based screening has advantages over in vitro assays, including the ability to consider cellular factors such as posttranslational modifications and cofactor concentrations^12–14^ and differences of membrane permeability of compounds into cells. *S*-*p*-bromobenzylglutathione (BBG), an analogue of GSH, is traditionally used inhibitor developed from in vitro assays^5,6^. Although BBG acts as a potent inhibitor of GLO1, the drug does not possess sufficient membrane permeability and must be used as the prodrug BBGC that requires activation in the targeted cells (Fig. S1). Despite the potency of the original drug, the prodrug requires relatively high concentrations (more than μM) to be effective in living cells^6^, and its activity is dependent on cellular esterase activities^15^. Recent in vitro HTS have characterized more compounds^16–19^, but a live cell-based HTS system for large-scale inhibitor screening is still desirable.

**Fig. 2:**
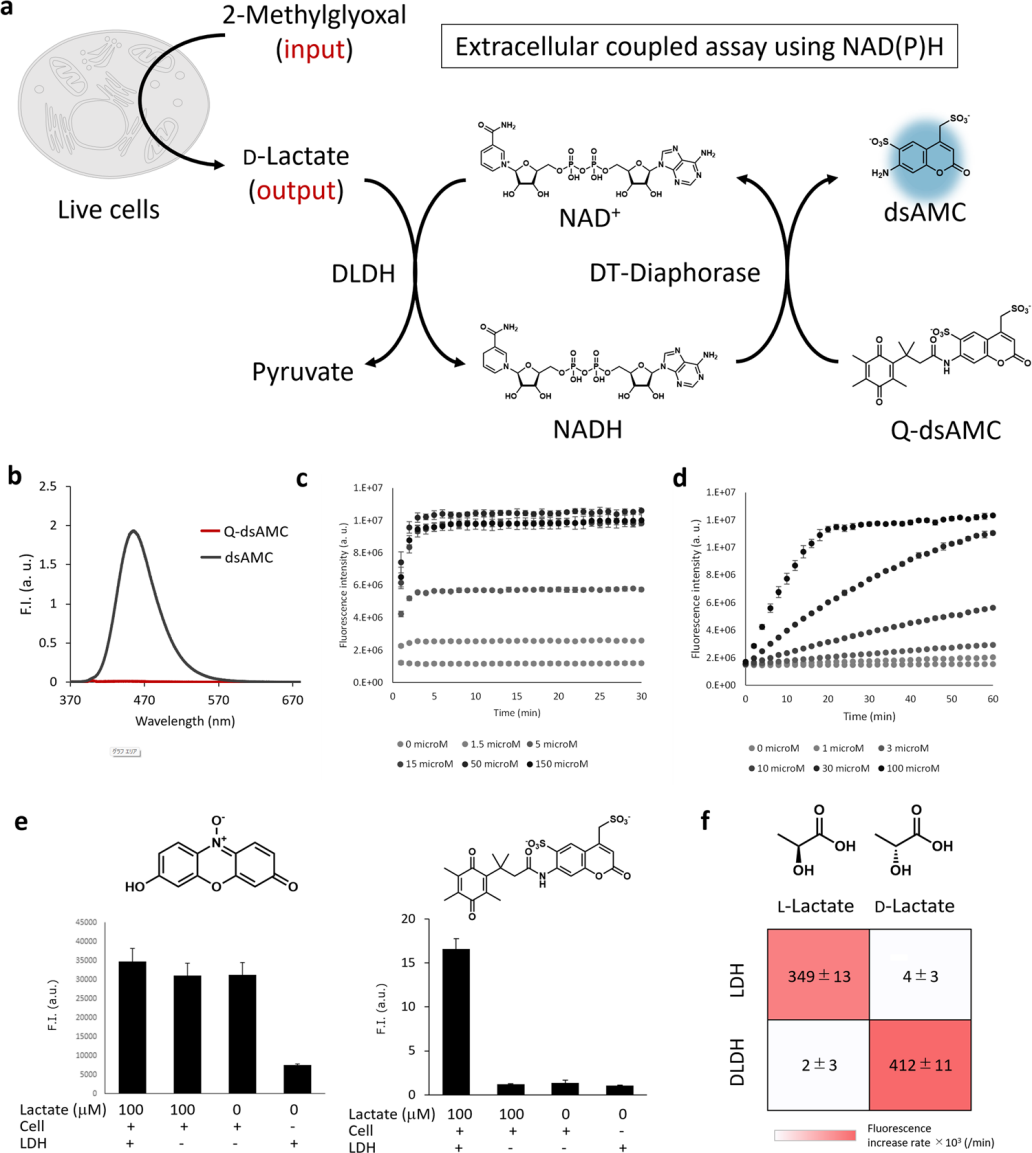
Design of fluorogenic probe for extracellular metabolite detection via NAD(P)H-coupled assay. **a** Structure of Q-dsAMC and the design of live cell-based coupled assay. **b** Fluorescence spectra (λ_ex._ = 360 nm) of 1 μM probes (Q-dsAMC and dsAMC) in PBS (pH 7.4) containing 0.1% DMSO as a co-solvent. **c** Fluorescence intensities of Q-dsAMC (10 mM) after mixing with NADH (0–150 μM), DT-diaphorase (5 μg/mL) in DPBS (pH 7.4) containing 0.1% CHAPS. Error bars represent S.D. (*n* = 4). **d** Fluorescence intensities of Q-dsAMC (10 μM) after mixing with d-lactate (0–100 μM), NAD^+^ (100 μM), DT-diaphorase (5 μg/mL), and d-lactate dehydrogenase (2.5 U/mL) in DPBS (pH 7.4). Error bars represent S.D. (*n* = 3). **e** Fluorescence intensities of (left) resazurin (1 μM) or Q-dsAMC (1 μM) after mixing with A549 cells (1 × 10^5^ cells/mL), l-lactate (0 or 100 μM), NAD^+^ (100 μM), DT-diaphorase (1 U/mL), and lactate dehydrogenase (LDH) (0 or 1 μg/mL) in DPBS (pH 7.4). **f** Fluorescence intensities of Q-dsAMC (10 μM) after mixing with NAD^+^ (100 μM), l-lactate or d-lactate (100 μM) with LDH or d-lactate dehydrogenase (DLDH) (5 μg/mL) in PBS (pH 7.4) containing 0.1% CHAPS and incubated for 10 min. The value is mean ± S. D. (*n* = 4).

We recently established the concept of live cell-based coupled assays for monitoring targeted metabolic pathways for live cell-based HTS^20,21^. In this metabolic pathway-oriented screening methodology, we specify the input and output metabolites, and the in situ detection of the extracellular metabolite (output) that is generated and released from cells can be used as a readout of the activity of the targeted metabolic pathways. Hence, the compounds that decrease the signals are characterized as hit compounds to control the targeted enzymes in the pathway (Fig. 2a).

## Results and discussion

The key factors for developing metabolic pathway-oriented screenings are: (1) selection of proper input and output metabolites, and (2) development of fluorogenic probes to detect the target metabolites exclusively in the extracellular space. Selective detection of output metabolites is realizable by coupled assays using NAD(P)H^20^. For live cell-based coupled assays, the assay system needs to be separated from the intracellular space that contain high concentration of NAD(P)H, so the probe should be retained in extracellular media throughout the assay.

For *factor 1*, the proper input was determined as MG, and the output was d-lactate generated downstream of the glyoxalase system (Fig. 1). Because we cannot expect a large quantity of d-lactate to be generated from the system, development of a proper detection system for *factor 2* is also important. d-Lactate can be used as a substrate of d-lactate dehydrogenase to generate NADH from NAD^+^, and NADH can be fluorescently detected using a fluorogenic-coupled assay. In a previous study, formazan derivatives were used to detect extracellular metabolites through the NADH-coupled assay^20^, but their reactivity to NADH was not sufficiently high, and we employed another strategy of fluorogenic NAD(P)H detection via an enzyme-coupled assay using DT-diaphorase.

DT-diaphorase uses NAD(P)H to reduce various electrophiles, among which permethylated quinone propionic acid is known to be a good reaction site for the enzyme^22^. There are known DT-diaphorase substrates for in vitro NAD(P)H detection^23,24^, but we needed a membrane impermeable probe for extracellular metabolite detection, and we prepared the probe based on 7-amino-4-methylcoumarin scaffold with two sulfonic acids (dsAMC). Introduction of sulfonic acid contributes to the increased hydrophilicity of the molecule so that the probe does not enter the cells to generate undesired signals via the reaction with intracellular NAD(P)H. Then, we attached the permethylated quinone propionic acid (Fig. 2a). The Q-dsAMC probe reacted rapidly with NADH in the co-presence of DT-diaphorase and exhibited fluorescence activation (Fig. 2b, S2). It reacted almost quantitatively with NADH within 10 min, and the assay was able to detect 100 nM NADH (Fig. 2c), which was barely detectable in the previous formazan-based probe (Fig. S3a)^20^. The system can detect the NADH generated from the oxidation of d-lactate to pyruvate by d-lactate dehydrogenase (Fig. 2d). Besides the target detectability, the important feature of the probe is to have sufficient hydrophilicity to avoid the entry into the cell in the live cell-based coupled assay. In order to test this, we have monitored l-lactate generated from live cells from the metabolism of glucose and compared the results with that of resazurin^25^, a present NAD(P)H detecting probe. Owing to its hydrophilicity, Q-dsAMC was able to detect extracellular metabolites under the presence of live cells, while resazurin entered the cells and failed to report the extracellular metabolites (Fig. 2e).

Then, we have developed the live cell-based coupled assay to monitor glyoxalase pathway activities in living tumor cells. For the conversion of the output metabolite d-lactate to fluorogenic signals, we used microbial d-lactate dehydrogenase that oxidate d-lactate using NAD^+ ^^26^. Since cells also generate and secrete l-lactate from glycolysis, it is important that the system exhibit sufficient selectivity to distinguish the enantiomers. We confirmed that DLDH and LDH were completely orthogonal system; DLDH showed more than 100 times selectivity toward d-lactate over l-lactate (Fig. 2f, S3b). Then, we applied the coupled assay to detect the glyoxalase activity in living cells monitored under the input MG. Various non-tumor and tumor cells from lung and colon origins were treated with or without MG. Without MG, only negligible levels of extracellular d-lactate were detected, whereas the addition of MG increased the signal (Fig. S4). In the following study, we focused on DMS114 and DMS273 cells that are derived from small cell lung cancer (SCLC). SCLC comprises approximately 15% of lung cancers^27^, but its malignancy and poor prognosis require the development of a novel way of treatment. The therapeutic potential of glyoxalase inhibitors for the treatment of SCLC was previously reported^6^, and we attempted to discover novel glyoxalase inhibitors via the live cell-based HTS using SCLC cells.

**Fig. 3:**
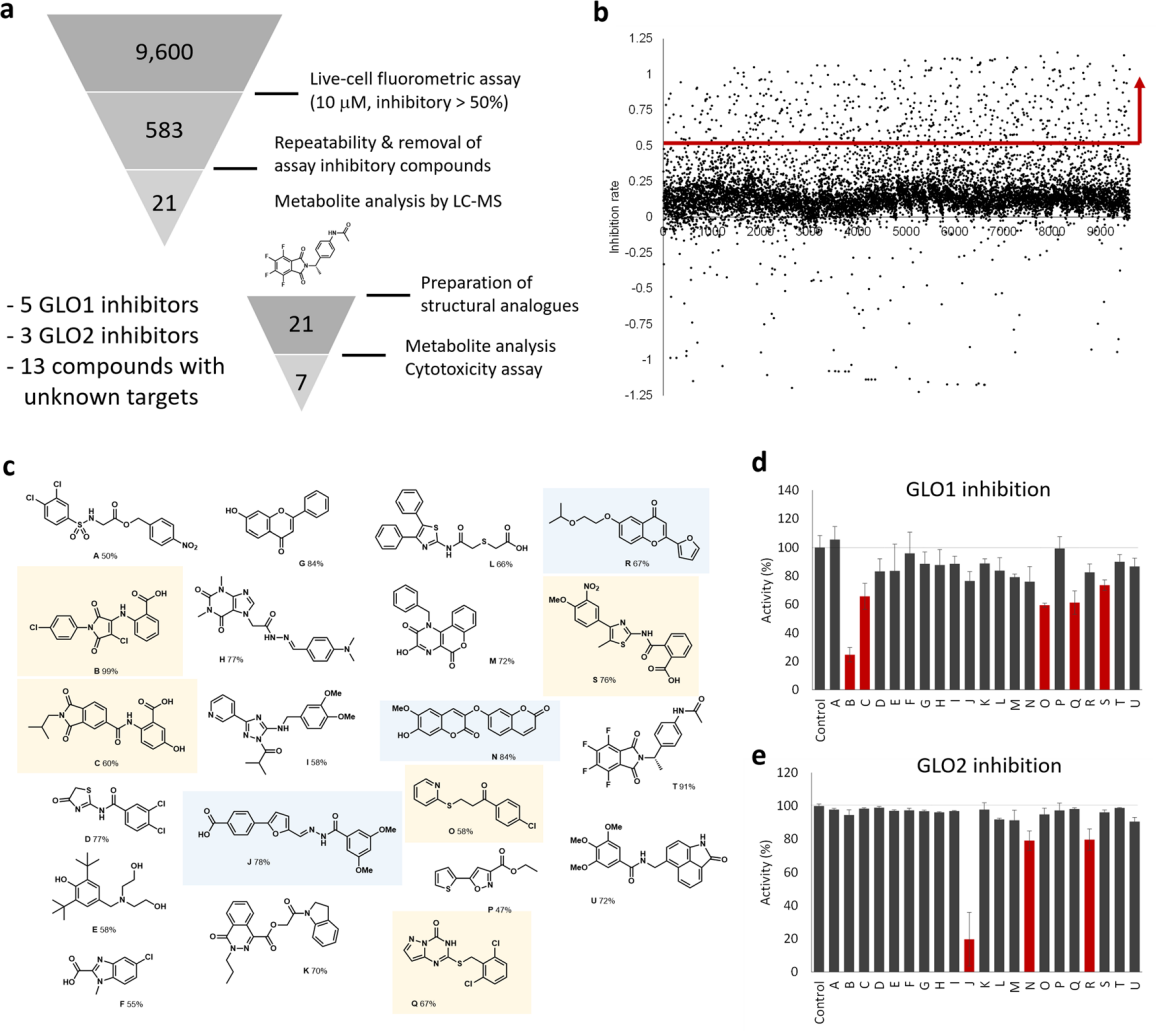
Screening of compounds that affect glyoxalase pathway of DMS114 cells. **a** Flow of screening. **b** Summary results of 1st screening. **c** Structures of hit compounds that blocked the glyoxalase pathway activity of DMS114 cells. Numbers indicates the inhibition % of the activity. Yellow highlighted compounds blocked GLO1 activity, and blue highlighted compounds blocked GLO2 activity. **d** Activities of recombinant GLO1 (0.1 μg/mL) after mixing with MG (100 μM), GSH (1 mM) and inhibitors (10 μM) in PBS (pH 7.4) and incubating for 10 min. The formation of SLG was monitored by LC-MS, and the percentage of the activity was calculated compared with conditions without inhibitors. Error bars represents S. D. (*n* = 3). Red bars indicate compounds with inhibition of >30% with *P* < 0.05 (Student’s *t*-test). **e** Activities of recombinant GLO2 (0.17 μg/mL) after mixing with SLG (100 μM) and inhibitors (10 μM) in PBS (pH 7.4) and incubating for 30 min. The consumption of SLG was monitored by LC-MS, and the percentage of the activity was calculated compared with conditions without inhibitors. Error bars represents S. D. (*n* = 3). Red bars indicate compounds with inhibition of >30% with *P* < 0.05 (Student’s *t*-test).

We optimized the assay conditions using DMS114 cells and confirmed that we can evaluate the efficacy of present drug BBGC in the screening platform (Fig. S5a). We were able to run 384-well plate-based fluorometric assays with sufficient robustness (*Z*’ = 0.69, Fig. S5b). With the optimized assay condition in hand, we performed HTS using a 9600-compound library stored in the Drug Discovery Initiative, The University of Tokyo (Fig. 3a). In the first screening, the compounds that blocked the fluorescence increase to less than 50% at 10 μM in live cell-based assay were selected as hit compounds (583 compounds were selected, Fig. 3b). In the second screening, the confirmation of the repeatability and the exclusion of the assay inhibitory compounds (i.e., compounds that showed the decreased signals by blocking d-lactate dehydrogenase or DT-diaphorase) were performed (Fig. S6). At this stage, we have evaluated the reproducibility of the assay by comparing the activities of compounds observed in the first and second screenings, and the good recovery was confirmed (*R*^2^ = 0.93, Fig. S6a). Many hit compounds in the first screening were revealed to be assay inhibitory compounds. By excluding the assay inhibitory compounds and compounds whose activities were variable between assays, we selected 21 compounds that blocked glyoxalase system of DMS114 cells (Fig. 3c, Table S3).

**Fig. 4:**
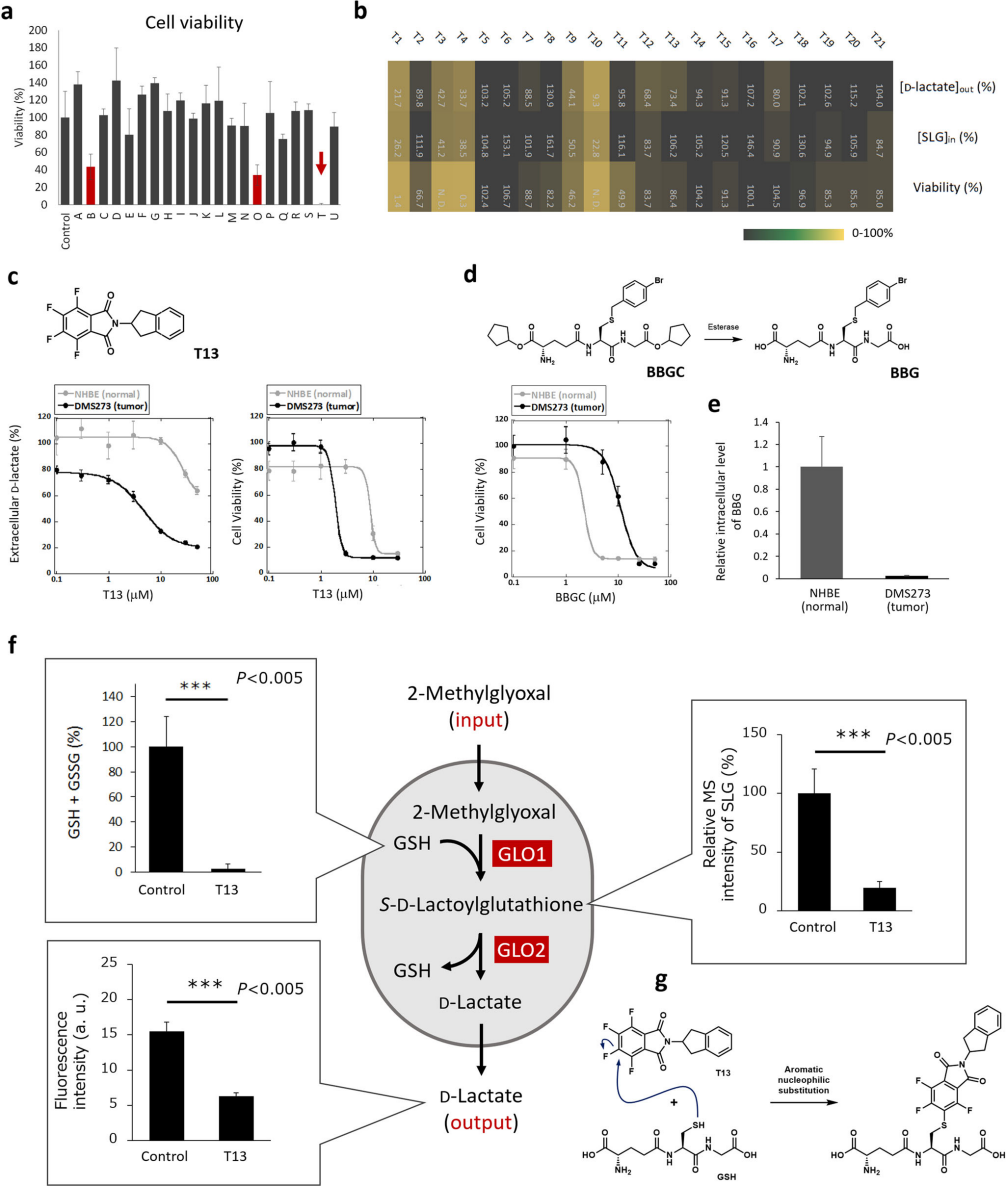
Understanding of the mechanism of compound T to affect live-cell glyoxalase activities. **a** Viability of DMS114 cells after treating with hit compounds (compounds **A**-**U**) in DPBS (pH 7.4) containing glucose (1 mM) and MG (100 μM) for 24 h. Error bars represent S.D. (*n* = 4). **b** Metabolic modulating activities (changes of intracellular SLG concentration and extracellular d-lactate concentration) and cytotoxicity (viability after 48 h) of pentafluorophthalimide analogues (**T1**-**T21**, 5 μM) toward DMS114 cells. **c** Dose-dependent inhibition of d-lactate formation and cell viability of **T13** against DMS273 (tumor, black line) and NHBE (non-tumor, grey line) cells. Error bars represent S.D. (*n* = 6). **d** Dose-dependent cytotoxicity of BBGC against DMS273 and NHBE cells. Error bars represent S.D. (*n* = 6). **e** Intracellular concentration of BBGC after incubating with 10 μM BBGC with DMS273 and NHBE cells. Error bars represent S.D. (*n* = 4). **f** Changes of metabolites with or without the addition of **T13** in living DMS273 cells. Error bars represent S.D. (*n* = 4). **g** Proposed mechanism of **T13** affecting cellular GLO1 activity.

The hit compounds were expected to be either (1) GLO1 inhibitors, (2) GLO2 inhibitors, or (3) compounds that affect cellular factors to control glyoxalase system. We classified them by monitoring the inhibition of recombinant GLO1 and GLO2 in in vitro assays. 5 out of 21 compounds were characterized to block the GLO1 activity (>30% inhibition at 10 μM, *P* < 0.05 compared with control, Figs. 3d), and 3 compounds were characterized to block GLO2 activity (>20% inhibition at 10 μM, *P* < 0.05 compared with control, Fig. 3e) at 10 μM. The inhibitory activities (inhibition %) were not completely parallel to those observed in live cell-based assay, which might have reflected the different cell permeabilities of inhibitors into cells. Among them, compound **B** was characterized as a potent GLO1 inhibitor, and it strongly blocked cellular glyoxalase system and showed strong cytotoxicity under the presence of MG (Fig. 4a). Most of other hit compounds that showed weaker inhibitory activity at 10 μM did not induce sufficient cell death, indicating that the residual glyoxalase activity from the weaker inhibitory activity was sufficient to eliminate MG.

Interestingly, another hit compound (Compound **T**), tetrafluorophthalimide, which was developed as a thalidomide analog^28^, showed a potent glyoxalase inhibitory activity and cytotoxicity (Figs. 3c, 4a), while this compound did not block either GLO1 or GLO2 (Fig. 3d, e). Therefore, it seemed to target other factors to control glyoxalase system in living cells, and we considered that understanding of the mechanism-of-action of the compound will lead to the identification of novel means of controlling MG pathway in tumor cells. The compounds have been reported to modify TGF-α signalling in tumor cells^28^, but as far as current biological knowledge covers, the effect on the MG metabolic pathway seemed to be independent of this mechanism of action. For the mechanism analysis, we firstly performed a structure-activity relationship study using an extended library of derivatives of compound **T** with varied amino groups of phthalimides and with different stereochemistry (**T1**–**T21**, Fig. 4b, S7a). Among 21 derivatives tested, seven compounds inhibited d-lactate generation (inhibition >25%) at 10 μM. All active compounds bare pentafluorophthalimide structure, so this scaffold seemed to be necessary for the activity (Fig. S7a). Inhibition of d-lactate generation (at 1 h) and cytotoxicity (at 48 h) were highly correlated (*R*^2^ = 0.71, Fig. S7b), indicating that the modulation of metabolic activities by the compounds was correlated to cell death-inducing activity. Among the compounds that showed activity in both DMS114 and DMS273 cells, we selected **T13** for further investigation of its mechanism of action, since other hit compounds such as **T1** and **T9** showed the cytotoxicity to non-tumor NHBE cells at lower concentration (Fig. S7c). **T13** exhibited inhibitory activity of d-lactate formation and cytotoxicity in DMS273 cells at comparable concentration ranges (IC_50_ = 4.6 μM, LC_50_ = 1.9 μM, Fig. 4c, Table S4). The compound showed the toxicity to non-tumor NHBE cells as well, but higher concentration was required (LC_50_ = 8.7 μM). BBGC required higher concentrations to function in tumor cells (LC_50_ = 10.9 μM), while non-tumor NHBE cells were killed at lower concentrations (LC_50_ = 2.2 μM, Fig. 4d). This discrepancy was because the active drug BBG accumulated more in NHBE cells owing to the differences in the metabolism of BBGC by cellular esterases (Fig. 4e).

Finally, the mechanism-of-action of the compound was characterized by determining the changes in the levels of intracellular metabolites after treating cells with the inhibitor; the target can be identified from the increase of upstream metabolites and the decrease of downstream metabolites in focused metabolite analysis^20^. As a result, a decrease in intracellular level of SLG, an intermediate of glyoxalase system, was observed, indicating that the compound acted on the generation of SLG (upstream) in the glyoxalase systems. We investigated the associated metabolites and discovered that the compound significantly decreased the intracellular GSH levels (Fig. 4f). Therefore, the effect of **T13** can be explained by its ability to reduce cellular GSH level, which acts as a co-substrate of GLO1, thereby lowering GLO1 activity in the cell. The mechanism by which **T13** reduces cellular GSH is not completely understood, but we discovered that the compound reacts with GSH as a co-substrate of glutathione *S*-transferase (GST; Fig. 4g, S8)^29^. The results imply that the perfluorinated thalidomide analogs^28^ act as a GST-mediated GSH-depleting agent to exhibit the cytotoxicity to tumor cells. Considering the importance of GSH in glyoxalase system, it is reasonable that GSH-depleting reagents efficiently shut down the glyoxalase system of cells. Cell death induced by the derivatives of compound **T** might be an outcome from combined effects downstream of GSH depletion, but shutting down of glyoxalase system may be one of the contributing factors. The underlying mechanism why the compound exhibited the selective cytotoxicity toward tumor cells over non-tumor cells are not fully understood, but it might have reflected the alterations of GST activities in the cells^30^, and the detailed study along this line is currently ongoing.

In conclusion, we established a metabolic pathway-oriented screening approach in the search for compounds that control the MG-metabolizing activity of live cells. The live cell-based assay afforded compounds that directly or indirectly block the glyoxalase activities in the targeted cells, and the mechanisms of the indirect inhibitors can be understood from the targeted metabolite analysis. The assay does not require genetic modification of the cells, and it can be widely applicable to cells (including primary culture cells) and organoids to search for compounds to control MG-metabolic pathways of targeted cells.

## Methods

### General materials in chemical synthesis

All chemicals used were of analytical grade and were purchased from Tokyo Chemical Industries, Fujifilm-Wako Pure Chemical Industries, or Merck. Recombinant GLO1 was purchased from R&D Biosystems (4959-GL, Lot #RHT0921121), recombinant GLO2 was purchased from R&D Biosystems (5944-GO, Lot #TKQ0321051), lactate dehydrogenase was purchased from Sigma-Aldrich (L2500, Lot #SLBT2408), d-lactate dehydrogenase was purchased from Toyobo (LCD-211, Lot #74580), and DT-diaphorase was purchased from Sigma-Aldrich (D1315-1MG, Lot #SLBX2524) and Toyobo (DAD-311, Lot #56210).

### Compound library

The library is composed of commercially available synthetic compounds prepared as part of a project by the Drug Discovery Initiative at the University of Tokyo. The full library is a drug-like diverse compound library of approximately 300,000 compounds (https://www.ddi.f.u-tokyo.ac.jp/en/). We performed the screening with a core library of 9600 compounds that cover the representative pharmacophores included in the library. Analogues of hit compounds, which were prepared according to the literature^28,31^, were identified from the library using structural similarity searches.

### Instruments used in chemical synthesis

NMR spectra were recorded using a JEOL JNM-ECZ400 spectrometer at 400 MHz for ^1^H NMR and 100 MHz for ^13^C NMR. Mass spectra (MS) were obtained using JEOL JMS-T100LP AccuTOF LC-plus 4 G (ESI).

Preparative HPLC was performed on an Inertsil ODS-3 (10.0 × 250 mm) column (GL Sciences Inc.) using an HPLC system composed of a pump (PU-2080, JASCO) and a detector (MD-2015). Preparative MPLC was performed on an Isolera One purification system (Biotage) equipped with a Biotage SNAP Ultra C18 column (for reverse phase separation) or on an MPLC system comprising a pump and detector (EPCLC AI-580S, Yamazen) and equipped with a silica gel column (silica gel 40 μm or Amino 40 μm, Yamazen) (for normal phase separation). LC-MS analysis was performed on an Acquity UPLC H-Class system (Waters) equipped with an Acquity UPLC BEH C18 1.7 μm (2.1 × 50 mm) column (Waters) and an MS detector (QDa or Xevo TQD, Waters).

### UV-visible absorption, fluorescence spectroscopy

UV-visible spectra were obtained using a UV spectrometer (UV-1850, Shimadzu). Fluorescence analysis was performed using a fluorometric spectrometer (F-7100; Hitachi). The slit width was 5.0 nm for both the excitation and emission. The photomultiplier voltage is 400 V.

### Enzyme activity measurement using a microplate reader

Fluorescence was measured in 384-well black plates (Greiner) using a microplate reader (2103 EnVision, PerkinElmer). Time course of fluorescence intensity (excitation and emission:335-375 nm/448-473 nm) was measured at 2 min intervals at 25 °C. The reaction rate was calculated at the initial velocity.

### Cell culture

H226, HCT116, DLD1, DMS114 and DMS273 cells were cultured in Roswell Park Memorial Institute medium (RPMI medium 1640 (1×), Gibco 11875-093), supplemented with 10% (v/v) fetal bovine serum, 1% (v/v) Penicillin-Streptomycin (Gibco, 15410-122). A549 and HT29 cells were cultured in Dulbecco’s Modified Eagle medium (DMEM medium (1×), Gibco 11885-084), supplemented with 10% (v/v) fetal bovine serum, 1% (v/v) Penicillin-Streptomycin (Gibco, 15410-122). NHBE cells were cultured in Bronchial Epithelial Cell Basal medium (BEBM, Lonza CC-3170), supplemented with Bronchial Epithelial Cell Growth Medium SingleQuots Supplements and Growth Factors (BEGM, Lonza, CC-4175). HCoEpic cells were cultured in Colonic Epithelial Cell Medium (CoEpiCM, ScienCell, #2951), supplemented with Colonic Epithelial Cell Growth Suppllement (CoEpiCGS, ScienCell, #2952). All cells were cultured in a humidified incubator under 5% CO_2_ in 95% air.

### Fluorometric assay for the screening

High-throughput screening (HTS) was performed against a chemical library of 9600 compounds at the Drug Discovery Initiative, The University of Tokyo. First, test compounds (50 nL of 2 mM DMSO stock solution) were placed in wells of a multiwell plate (Corning No. 3677) using a POD Automation Platform (Labcyte). Then, 10 μL of DMS114 cells (4 × 10^5^ cells/mL) in 100 μM 2-methylglyoxal in DPBS (pH 7.4) was added to all wells using a Multi-Drop combi (Thermo Fischer Scientific). After incubation at r.t. for 1 h, 10 μL probe solution (SQC (2 μM), DT-diaphorase (2 U/mL), d-Lactate dehydrogenase (2 U/mL), NAD^+^ (200 mM) in DPBS (pH 7.4)) was dispensed into the wells, and the plates were incubated at r.t. for 20 min. The fluorescence intensity of dsAMC was measured (Ex./Em. = 380/490 nm) using a microplate reader (PHERAster, BMG LABECH). Negative or positive control wells (i.e., including 100 or 0 μM MG, *n* = 16) were also prepared for all plates. For the screening of assay inhibitory compounds, the cells were incubated with 20 μM d-lactate instead of 200 μM MG, and the fluorescence assay was performed in the same condition.

### Cell viability assay

NHBE, DMS114, or DMS273 cells were seeded at 5 × 10^3^ cells per well (96 well plate) in BEGM, RPMI1640, or DPBS (pH 7.4) containing 1 mM glucose and 100 μM MG with/without inhibitor (0.3, 1 or 10 μM test compound, *n* = 3 for each). After 24 h (for DPBS containing glucose and MG) or 48 h (for BEGM or RPMI1640) incubation at 37 °C, CCK-8 assay was performed by adding 10 μL CCK-8 solution (Cell Counting Kit-8, Donjin) to each well. Absorbance at 450 nm was measure by a microplate reader (Envision). The calibration curve was calculated using the KaleidaGraph software, and following equation was used (m1-m4 were variables). *y* = m1 + (m2 − m1)/(1 + (x/m3)^m4); m1 = max response, m2 = min response, m3 = LC_50_ or EC_50_, m4 = Hill’s coefficient.

### Measurement of metabolites of methylglyoxal metabolic pathway

DMS273 cells were seeded at 4 × 10^5^ cells per tube in 100 μM MG and the inhibitor (0 or 10 μM) in DPBS (pH 7.4) (*n* = 4). After 2 h incubation at 37 °C, sample was centrifuged, and supernatant was collected. Extracellular d-lactate was measured by analysis of the supernatant with Q-dsAMC. After collection of supernatants, the cells were washed with DPBS (pH 7.4) and lysed by vortexing with MeOH/H_2_O (80/20, v/v). The lysate was centrifuged, and the supernatant was collected. Intracellular *S*-D-lactoylglutathione (SLG) was measured by analysis of the cell extract with LC-MS/MS. For detection of intracellular GSH, the cell extract (15 μL) was mixed with 15 μL 5 mM Dansyl-Cl in MeCN and 15 μL 100 mM borate buffer (pH 9.1) at r.t. for 30 min, then quenched by adding 15 μL MeCN containing 10% formic acid. The mixture was subjected to LC-MS/MS. LC-MS/MS analysis was performed on an Acquity UPLC H-Class system (Waters) equipped with an Acquity UPLC BEH C18 1.7 μm (2.1 × 50 mm) column (Waters) and an MS detector (Xevo TQD, Waters). Detection was performed in the positive mode. For SLG, the fragment of *m/z* = 366.2 > 170.1 was used (cone voltage: 25 V, collision voltage: 25 V). For GSH, the fragment *m/z* = 367.2 > 170.1 was used (cone voltage: 25 V, collision voltage: 25 V).

### Evaluation of the inhibitory activity of GLO1 activity in in vitro

GSH (1 mM), MG (100 μM) and inhibitor (10 μM) were mixed for 15 min at r.t. and GLO1 enzyme (1 μg/mL) was added. After 5 min incubation at r.t., the mixture was quenched by MeOH and measured by LC-MS/MS. Detection was performed in the positive mode. For SLG, the fragment of *m/z* = 366.2 > 170.1 was used (cone voltage: 25 V, collision voltage: 25 V).

### Preparation of recombinant glutathione *S*-transferase (GST) and reaction of compound with GSH

Recombinant N-terminally hexahistidine tagged his-GSTP1^32^ was prepared from *Escherichia coli* BL21(DE3)pLysS transformed with pCOLD-I/GSTP1 and purified using Ni-NTA Agarose (QIAGEN) and the aliquot was kept at −80 °C until use. For the identification of GSH adduct of compound, compound T13 (100 μM) was reacted with GSH (1 mM) in DMSO for 1 h, and the reaction mixture was directly injected into LC-MS/MS for monitoring at 254 nm absorbance and MS (*m/z* = 100-700, positive mode), and the unique product was identified from mass spectrum. MRM condition was constructed from the same reaction solution mixture (*m/z* = 623.3 > 117.3, 391.1 (ESI^+^)). For testing the reactivity of compound T13 with GST, T13 (10 μM) was reacted with or without GSH (1 mM) and GSTP1 (10 μg/mL) at 37˚C for 1 h, and the product was analyzed by monitoring MRM chromatograms for T13 and T13-GSH.

### Reporting summary

Further information on research design is available in the Nature Portfolio Reporting Summary linked to this article.

## Supplementary information

Supplementary Information

Description of Additional Supplementary Files

Supplementary Data 1

Reporting Summary

## Data Availability

The datasets generated during and/or analysed during the current study are available from the corresponding author on reasonable request. All spectral data are available in Supplementary Data 1.
