# Supplementary material for: Development of pathway-oriented screening to identify compounds to control 2-methylglyoxal metabolism in tumor cells
Source: Commun Chem. 2023 Apr 13;6:68. doi: 10.1038/s42004-023-00864-y (PMC10102174; doi:10.1038/s42004-023-00864-y)
Supplement: Supplementary file 1 — Supplementary Information [file 42004_2023_864_MOESM1_ESM.pdf]

Supplementary Information

Development of pathway-oriented screening to identify compounds to control 2-methylglyoxal metabolism in tumor cells

Kouichi Yanagi, Toru Komatsu, Yuuta Fujikawa, Hirotatsu Kojima, Takayoshi Okabe, Tetsuo Nagano, Tasuku Ueno, Kenjiro Hanaoka and Yasuteru Urano

Contents

Supplementary data for preparation of compounds
Supplementary tables
Supplementary figures
Supplementary references

Supplementary data for preparation of compounds

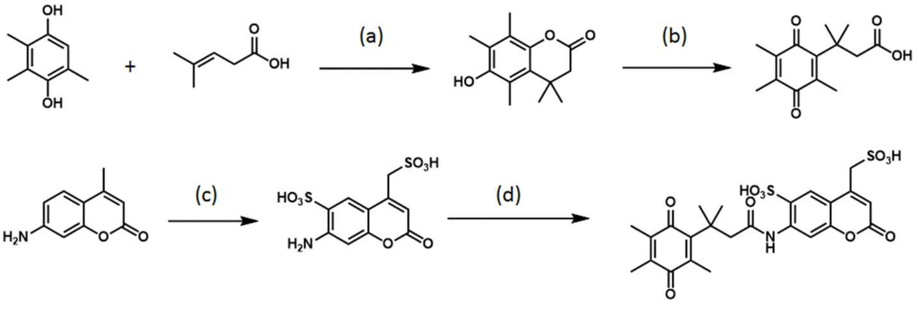

Scheme S1. Preparation of Q-dsAMC. (a) $MeSO_3H$, 85°C, Yield: quant. (b) NBS, $MeCN/H_2O$, r.t., Yield: 76.7% (c) $ClSO_3H$, 0°C to 120°C, Yield: 13.5% (d) 3-methyl-3-(2,4,5-trimethyl-3,6-dioxo-cyclohexa-1,4-dienyl)-butyric acid, HATU, DIEA, r.t., Yield: 6.1%.

Preparation of 6-hydroxy-4,4,5,7,8-pentamethyl-3,4-dihydrocoumarin (1)

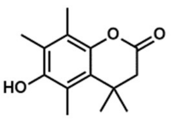

Trimethylhydroquinone (3.04 g, 20.0 mmol) and 3-methylcrotonic acid (2.20 g, 22.0 mmol) were dissolved in $MeSO_3H$ (15 mL), and the solution was stirred at 85°C for 6 h under Ar. Then, the mixture was cooled to r.t. and the product was extracted with AcOEt and washed with sat.$NaHCO_3$ aq.. The organic layer was evaporated to dryness and pumped up to afford

1 (4.70 g, 20.1 mmol, quant yield).
^{1}H NMR (400 MHz, $CDCl_3$): δ 1.44 (s, 6H), 2.17 (s, 3H), 2.20 (s, 3H), 2.34 (s, 3H), 2.53 (s, 2H), 4.73 (s, 1H).
^{13}C NMR (100 MHz, $CDCl_3$): δ 12.4, 12.7, 14.6, 26.7, 27.8, 35.6, 46.2, 119.0, 121.9, 123.5, 128.3, 143.6, 148.9, 169.0.
HRMS (ESI$^+$): Calcd. for $[M+H]^+$ 235.13342, Found 235.13197 (-1.5 mmu).

Preparation of 3-methyl-3-(2,4,5-trimethyl-3,6-dioxo-cyclohexa-1,4-dienyl)-butyric acid (2)

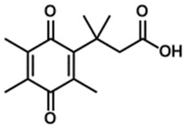

Compound **1** (4.70 g, 20.1 mmol) was dissolved in MeCN/H_2O (6/12 mL), and then N-bromosuccinimide (NBS, 3.86 g, 21.7 mmol) was added. The mixture was stirred at r.t. for 2 h, then the mixture was extracted with AcOEt/2N HCl aq. and evaporated to dryness. The residue was purified by MPLC (silica, eluent, 50% CH_2Cl_2/hexane (0 min) to 100% CH_2Cl_2/hexane (9 min)) to afford **2** (3.86 g, 15.4 mmol, 76.7 % yield).
^{1}H NMR (400 MHz, $CDCl_3$): δ 1.33 (s, 6H), 1.82 (s, 3H), 1.85 (s, 3H), 2.04 (s, 3H), 2.91 (s, 2H), 10.94 (s, 1H).
^{13}C NMR (100 MHz, $CDCl_3$): δ 12.1, 12.5, 14.3, 28.8, 37.9, 47.3, 138.3, 139.0, 143.0, 152.1, 178.7, 187.4, 190.8.
HRMS (ESI$^-$): Calcd. for $[M-H]^-$ 249.11268, Found 249.10864 (-4.1 mmu).

Preparation of 7-amino-2-oxo-4-(sulfomethyl)-2*H*-chromene-6-sulfonic acid (3, dsAMC)

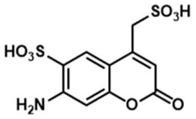

$ClSO_3H$ (2 mL) was added slowly to 7-amino-4-methylcoumarin (554 mg, 3.16 mmol) at 0°C and the mixture was stirred at 120°C for 4 h. The mixture was cooled to 0°C and quenched with H_2O and neutralized with 2N NaOH aq. The residue was purified by HPLC (eluent, 4% CH_3CN/0.1% TEAA aq. (0 min) to 80% CH_3CN/0.1% TEAA aq. (25 min); flow rate = 5.0 mL/min) and desalted with H_2O to afford **3** as a triethylamine salt (230 mg, 0.43 mmol, 13.5 % yield).
^{1}H NMR (400 MHz, CD_3OD): δ 4.19 (s, 2H), 6.18, (s, 1H), 6.63 (s, 1H), 8.20 (s, 1H).
^{13}C NMR (100 MHz, CD_3OD): δ 52.5, 100.9, 108.2, 111.4, 125.5, 126.4, 149.3, 149.4, 156.5,

162.0.
HRMS (ESI$^-$): Calcd. for $[M-H]^-$ 333.96913, Found 333.96614 (-3.0 mmu).

Preparation of compound 4 (Q-dsAMC)

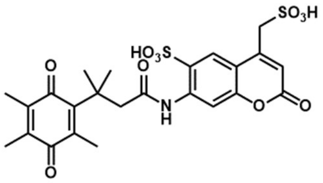

Compound **3** (54 mg, 0.10 mmol), compound **2** (73.5 mg, 0.29 mmol), HATU (108 mg, 0.28 mmol) and DIEA (81 μL, 0.47 mmol) were dissolved in MeCN (1 mL). The mixture was stirred at r.t. for 16 h, and evaporated to dryness. The residue was purified by HPLC (eluent, 4% CH_3CN/0.1% TEAA aq. (0 min) to 80% CH_3CN/0.1% TEAA aq. (25 min); flow rate = 5.0 mL/min) and desalted with H_2O to afford **4** as a triethylamine salt (4.7 mg, 0.0061 mmol, 6.1 % yield).
^1H NMR (400 MHz, DMSO-d_6): δ 1.38 (s, 6H), 1.92 (s, 3H), 1.95 (s, 3H), 2.05 (s, 3H), 2.95 (s, 2H), 3.86 (s, 2H), 6.25 (s, 1H), 8.08 (s, 1H), 8.09 (s, 1H), 10.61 (s, 1H).
HRMS (ESI$^-$): Calcd. for $[M+Na-2H]^-$ 588.06102, Found 588.06169 (+0.7 mmu).

Supplementary Tables

Table S1. Reports about the upregulation of glyoxalase system activities in various tumor cells.

Cancer	Upregulation	Cell growth	Reference
Gastric cancer	GLO1 gene	Inhibition by BBGCD (*in cellulo*)	*Oncogene*, **2015**, *34*, 1196-1206.
Breast cancer	GLO1 mRNA and protein	Inhibition by siRNA (*in cellulo*)	*Int. J. Clin. Exp. Pathol.*, **2017**, *10*, 10852-10862.
Prostate cancer	GLO1 activity	Inhibition by BBGCD (*in cellulo* and *in vivo*)	*Clin. Cancer Res.*, **2001**, *7*, 2513-2518.
Melanoma	GLO1 mRNA	Inhibition by siRNA (*in cellulo*)	*Melanoma Res.*, **2010**, *20*, 85-96.
Hepatocellular carcinoma	GLO1 mRNA	Inhibition by shRNA (*in cellulo* and *in vivo*)	*Int. J. Clin. Exp. Pathol.*, **2014**, *7*, 2079-2090.
Lung cancer (NSCLC, SCLC)	GLO1 activity	Inhibition by BBGCD (*in cellulo* and *in vivo*)	*Clin. Cancer Res.*, **2001**, *7*, 2513-2518.
Leukimic stem cells (Bcr-Abl^{+})	GLO1 activity	Inhibition by BBGCD (*in cellulo*)	*Cell Death Differ.*, **2010**, *17*, 1211-1220.
Breast cancer (Triple negative)	GLO1 activity	Not Determined	*Oncotarget*, **2012**, *5*, 5472-5482.
Pancreatic cancer	GLO1 protein	Not Determined	*Anticancer Res.*, **2012**, *32*, 3219-3222.
Leukemia (apoptosis-resistant UK711 and UK110 cells)	GLO1 mRNA	enhanced etoposide-induced apoptosis by cotreatment with BBGCD (*in cellulo*)	*Blood*, **2000**, *95, 3214-3218.*

Table S2. List of currently available assays to monitor glyoxalase activities[1].

	**Detection of metabolite**	**Method**	**Throughput**	**Assay**
**Glyoxalase 1 activity**	*S*-D-lactoylglutathione	Spectrophotometric assay at 240 nm	High	*In vitro*
		LC-MS	Low	*In vitro*
**Glyoxalase 2 activity**	*S*-D-lactoylglutathione	Spectrophotometric assay at 240 nm	High	*In vitro*
		LC-MS	Low	*In vitro*
	GSH	LC-MS	Low	*In vitro*
	D-lactate	LC-MS	Low	*In vitro*

Table S3. Names of hit compounds and the inhibitory activity of glyoxalase system of DMS114 cells.

Compound	Name	Activity
**A**	4-nitrobenzyl ((3,4-dichlorophenyl)sulfonyl)glycinate	50%
**B**	2-((4-chloro-1-(4-chlorophenyl)-2,5-dioxo-2,5-dihydro-1*H*-pyrrol-3-yl)amino)benzoic acid	99%
**C**	5-hydroxy-2-(2-isobutyl-1,3-dioxoisoindoline-5-carboxamido)benzoic acid	60%
**D**	3,4-dichloro-N-(4-oxo-4,5-dihydrothiazol-2-yl)benzamide	77%
**E**	2,2'-((3,5-di-tert-butyl-4-hydroxybenzyl)azanediyl)bis(ethan-1-ol)	58%
**F**	5-chloro-1-methyl-1H-benzo[*d*]imidazole-2-carboxylic acid	55%
**G**	7-hydroxy-2-phenyl-4*H*-chromen-4-one	84%
**H**	(*E*)-2-(1,3-dimethyl-2,6-dioxo-1,2,3,6-tetrahydro-7*H*-purin-7-yl)-*N*'-(4-(dimethylamino)benzylidene)acetohydrazide	77%
**I**	1-(5-((3,4-dimethoxybenzyl)amino)-3-(pyridin-3-yl)-1*H*-1,2,4-triazol-1-yl)-2-methylpropan-1-one	58%
**J**	(*E*)-4-(5-((2-(3,5-dimethoxybenzoyl)hydrazineylidene)methyl)furan-2-yl)benzoic acid	78%
**K**	2-(indolin-1-yl)-2-oxoethyl 4-oxo-3-propyl-3,4-dihydrophthalazine-1-carboxylate	70%
**L**	2-((2-((4,5-diphenylthiazol-2-yl)amino)-2-oxoethyl)thio)acetic acid	66%
**M**	1-benzyl-3-hydroxy-2*H*-chromeno[3,4-*b*]pyrazine-2,5(1*H*)-dione	72%
**N**	7-hydroxy-6-methoxy-3-((2-oxo-2*H*-chromen-7-yl)oxy)-2*H*-chromen-2-one	84%
**O**	1-(4-chlorophenyl)-3-(pyridin-2-ylthio)propan-1-one	58%
**P**	ethyl 5-(thiophen-2-yl)isoxazole-3-carboxylate	47%
**Q**	2-((2,6-dichlorobenzyl)thio)pyrazolo[1,5-*a*][1,3,5]triazin-4(3*H*)-one	67%
**R**	2-(furan-2-yl)-6-(2-isopropoxyethoxy)-4*H*-chromen-4-one	67%
**S**	2-((4-(4-methoxy-3-nitrophenyl)-5-methylthiazol-2-yl)carbamoyl)benzoic acid	76%
**T**	(*S*)-*N*-(4-(1-(4,5,6,7-tetrafluoro-1,3-dioxoisoindolin-2-yl)ethyl)phenyl)acetamide	91%
**U**	3,4,5-trimethoxy-*N*-((2-oxo-1,2-dihydrobenzo[*cd*]indol-6-yl)methyl)benzamide	72%

Table S4. Parameters determined from the calibration curves of **Figure 4c** and **4d.**

Figure 4c left (Extracellular D-lactate, NHBE cells)

y=m1+(m2-m1)/(1+(x/m3)^m4);		
Parameters	Value	Errors
m1	56.628	18.835
m2	105.13	2.7788
m3	26.343	9.8328
m4	2.7442	2.2563
Chi-squared	88.365	NA
R	0.97594	NA

Figure 4c left (Extracellular D-lactate, DMS273 cells)

y=m1+(m2-m1)/(1+(x/m3)^m4);		
Parameters	Value	Errors
m1	19.817	2.1699
m2	77.934	1.4693
m3	4.6432	0.49594
m4	1.5385	0.22527
Chi-squared	11.038	NA
R	0.99859	NA

Figure 4c right (Cell viability, NHBE cells)

y=m1+(m2-m1)/(1+(x/m3)^m4);		
Parameters	Value	Errors
m1	15.034	4.3465
m2	81.724	2.115
m3	8.7348	102.35
m4	8.8654	769.14
Chi-squared	53.677	NA
R	0.99477	NA

Figure 4c right (Cell viability, DMS273 cells)

y=m1+(m2-m1)/(1+(x/m3)^m4);		
Parameters	Value	Errors
m1	11.821	1.3531
m2	98.114	1.1047
m3	1.8677	0.2503
m4	6.8326	1.8278
Chi-squared	10.979	NA
R	0.99956	NA

Figure 4d (Cell viability, NHBE cells)

y=m1+(m2-m1)/(1+(x/m3)^m4);		
Parameters	Value	Errors
m1	13.638	0.16197
m2	90.804	0.27746
m3	2.198	0.11052
m4	5.4832	0.34062
Chi-squared	0.15397	NA
R	0.99999	NA

Figure 4d (Cell viability, DMS273 cells)

y=m1+(m2-m1)/(1+(x/m3)^m4);		
Parameters	Value	Errors
m1	5.7547	6.2396
m2	101.02	3.8228
m3	10.873	1.1907
m4	2.8271	0.85925
Chi-squared	60.217	NA
R	0.99676	NA

Supplementary Figures

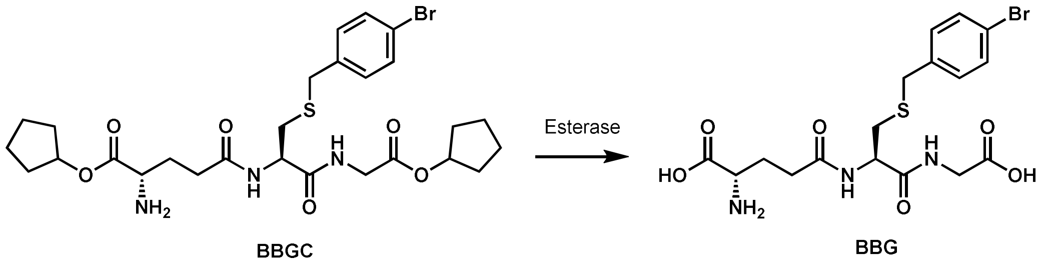

Figure S1. Activation of BBGC to BBG.

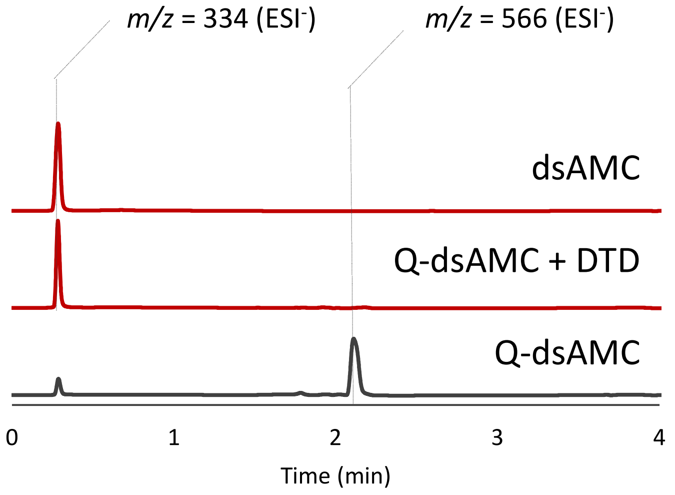

Figure S2. Reaction of Q-dsAMC with DT-diaphorase. LC chromatograms (λ = 320 nm) of Q-dsAMC (10 μM) after reacting with or without NADH (30 μM) and DT-diasphorase (1 U/mL) for 1 h. dsAMC is a reference product (10 μM).

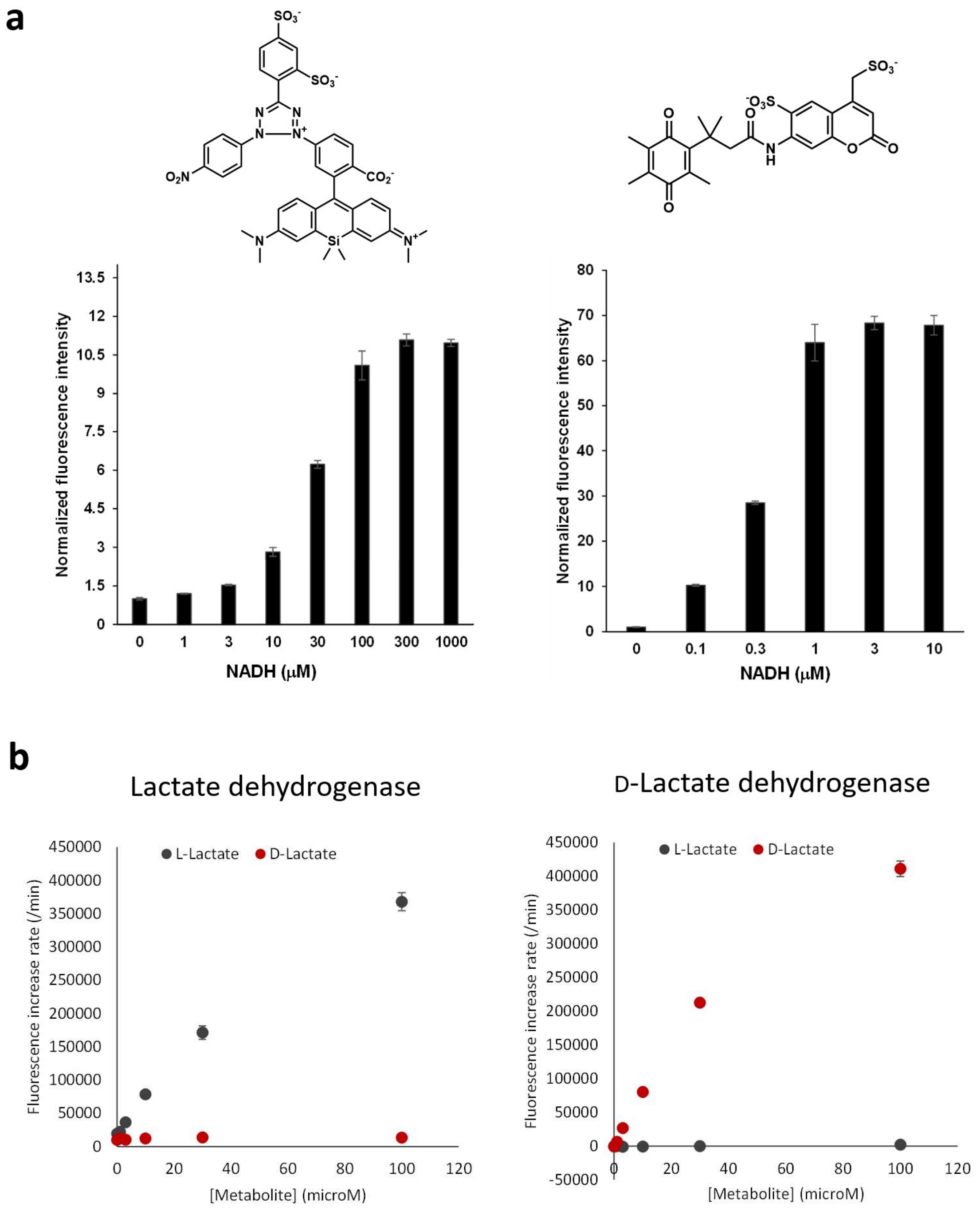

Figure S3. Characterization of Q-dsAMC in comparison with conventional probes used in live cell-based coupled assays. (a) Normalized fluorescence intensities of (left) SiRWST-3 (1 μM) after mixing with NADH (0-1000 μM) and 1-methoxy-PMS (10 μM) in DPBS (pH 7.4) or (right) Q-dsAMC (1 μM) after mixing with NADH (0-30 μM), DT-diaphorase (1 U/mL) in DPBS (pH 7.4). Error bars represent S.D. (n = 3). (b) Fluorescence intensities of Q-dsAMC (10 μM) after mixing with NAD^+ (0-100 μM), L-lactate or D-lactate (0-100 μM) with lactate dehydrogenase (left) or D-lactate dehydrogenase (right) (5 μg/mL) in PBS (pH 7.4) containing 0.1% CHAPS and incubated for 10 min. Error bars represent S. D. (n = 4).

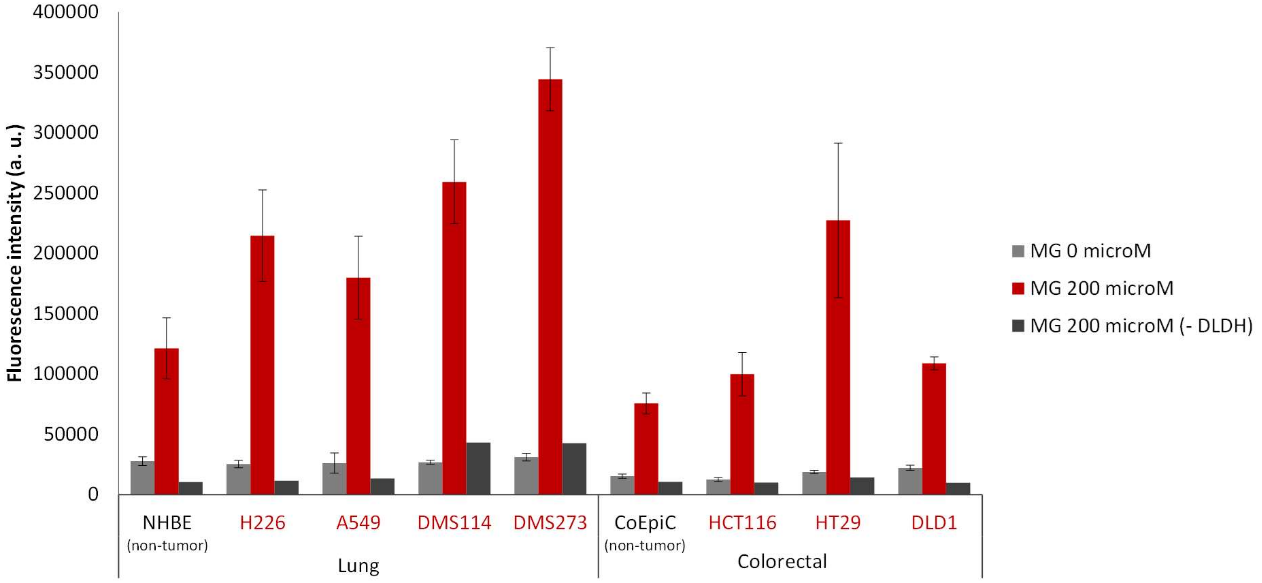

Figure S4. Detection of glyoxalase pathway activities in various tumor and non-tumor cells from lung and colon. Cells are incubated in DPBS (pH 7.4) containing 0 or 200 μM 2-MG with or without D-lactate dehydrogenase (DLDH, 1 U/mL). Cells (2×10^3 cells) were incubated with MG (0 or 200 μM) in DPBS for 1 h at 37°C. After incubation, probe solution was added and the fluorescence intensity was measured by plate reader. Error bars represent S.D. (n = 4).

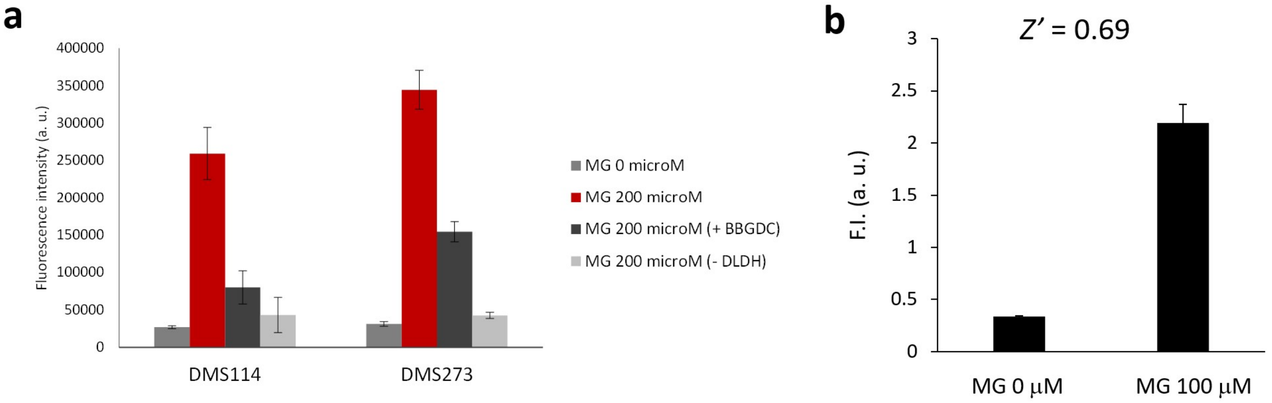

Figure S5. Validation of screening conditions. (a) Cells are incubated in DPBS (pH 7.4) containing 0 or 200 μM 2-MG with or without D-lactate dehydrogenase (DLDH, 1 U/mL) and with or without BBGC (50 μM). Error bars represent S.D. (n = 4). (b) Calculation of *Z*′-value. DMS114 cells (2×10^3 cells) were incubated with MG (0 or 100 μM) in DPBS for 2 h at r.t.. After incubation, the equal amount of probe solution was added and the fluorescence intensity was measured by plate reader (Ex./Em.= 355/460 nm). Probe solution: Q-dsAMC (2 μM), NAD^+ (200 μM), DT-diaphorase (2 U/mL), DLDH (2 U/mL) in DPBS. Error bars represent S.D. n = 16 (treatment with MG 0 μM), n = 336 (treatment with MG 100 μM).

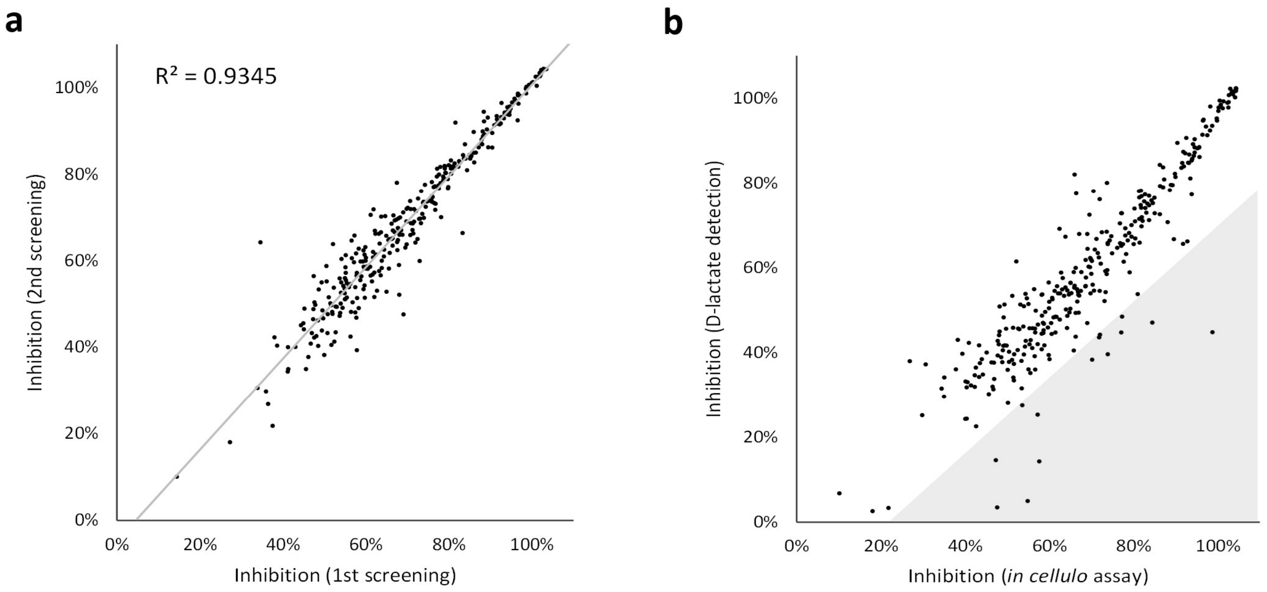

Figure S6. Repeatability of assay. (a) The results of 583 compounds selected as hit in 1st screening are shown. The horizontal axis indicates the inhibitory activity observed in 1st screening and the vertical axis indicates the inhibitory activity observed in 2nd screening. (b) Summary results of 2nd screening. The horizontal line indicated the inhibition in the live cell-based assay, and the vertical line indicated the inhibitory activity against D-lactate detection system. The compouds that are in gray triangle was chosen as hit comounds.

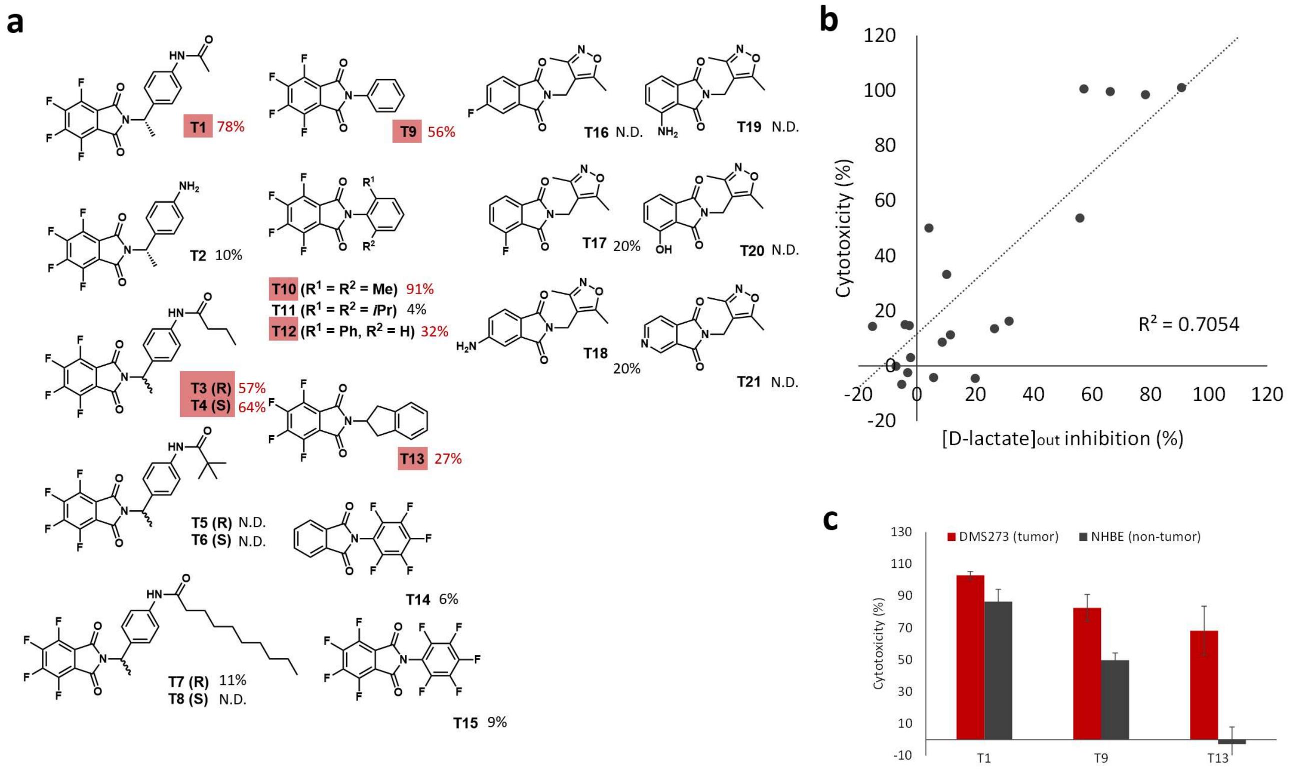

Figure S7. Glyoxalase pathway inhibitory activities and cytotoxicity of analogues of compound **T**. (a) Structures of analogues of compound **T** (**T1**-**T21**) and their glyoxal metabolic pathway modulatory activities (changes of extracellular D-lactate concentration) as shown in **Figure 4b**. Correlation of metabolic modulating activities (horizontal) and cytotoxicity (viability after 48 h, vertical) of **T1**-**T21** for DMS114 cells. (c) Cytotoxicity of compound **T1**, **T9** and **T13** toward DMS273 cells and NHBE cells. Error bars represent S.D. (n = 3).

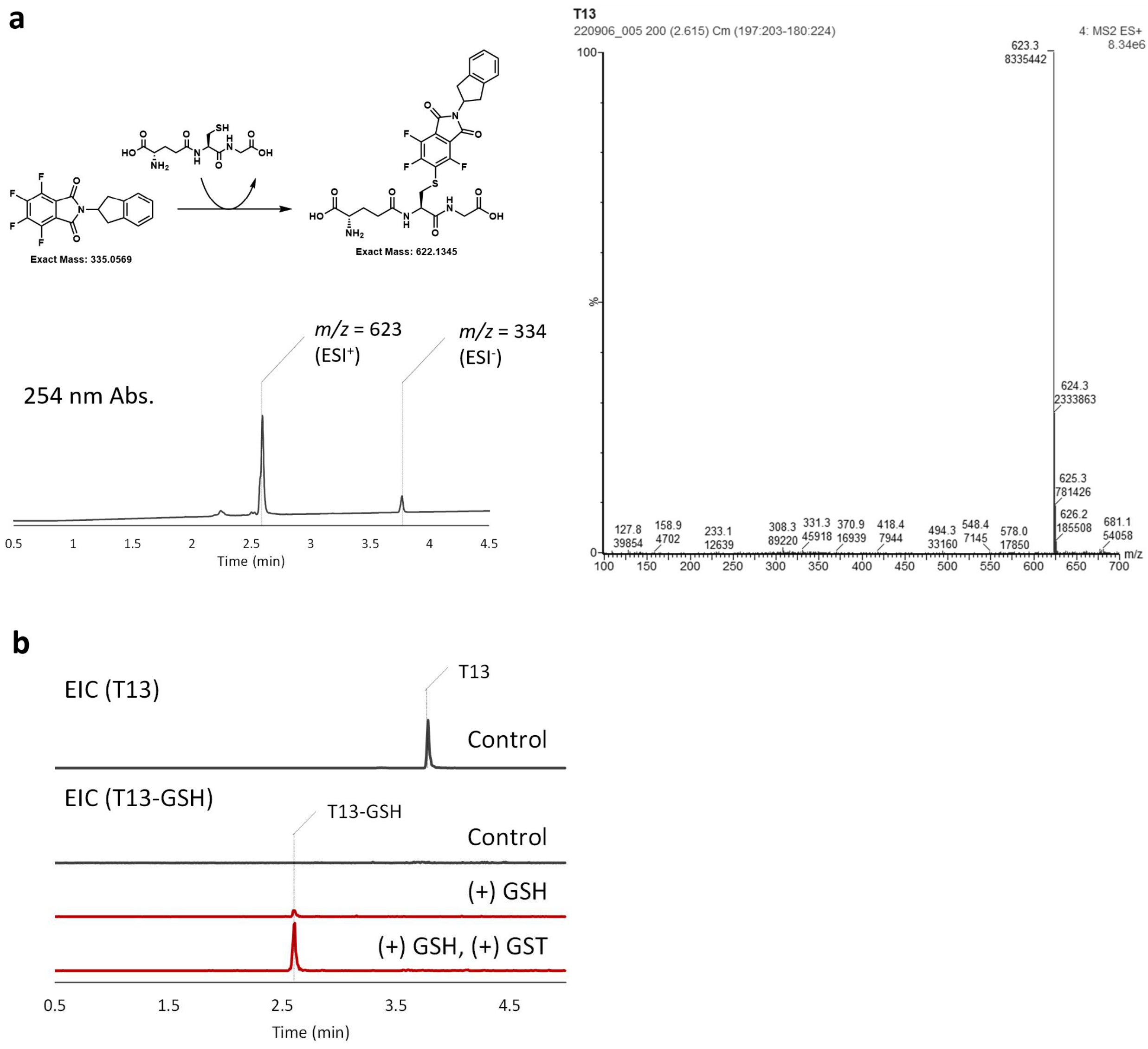

Figure S8. Tetrafluorophthalimides as GST-dependent GSH reacting reagents. (a) (left) LC-MS chromatogram of **T13** (100 μM) reacting with GSH (1 mM) in DMSO for 1 h. (right) Mass spectrum (ESI+) observed at 2.6 min. (b) Multiple reaction monitoring (MRM) chromatograms of **T13** (10 μM) reacting with or without GSH (1 mM) and glutathione *S*-transferase Pi (10 μg/mL) at 37°C for 1 h. MRM conditions were constructed for **T13** (*m/z* = 336.1 > 117.1 (ESI+)) and **T13** reacted with glutathione in DMSO (*m/z* = 623.3 > 117.3, 391.1 (ESI+)).

Supplementary References

1 N. Rabbani, M. Xue and P. J. Thornalley, *Clin. Sci.*, 2016, **130**, 1677–1696.
