# Supplementary material for: Development of pathway-oriented screening to identify compounds to control 2-methylglyoxal metabolism in tumor cells
Source: Commun Chem. 2023 Apr 13;6:68. doi: 10.1038/s42004-023-00864-y (PMC10102174; doi:10.1038/s42004-023-00864-y)
Supplement: Supplementary file 2 — Description of Additional Supplementary Files [file 42004_2023_864_MOESM2_ESM.pdf]

Description of Additional Supplementary File

File name: Supplementary Data 1

Description: Spectral Data
