# Supplementary material for: Development of pathway-oriented screening to identify compounds to control 2-methylglyoxal metabolism in tumor cells
Source: Commun Chem. 2023 Apr 13;6:68. doi: 10.1038/s42004-023-00864-y (PMC10102174; doi:10.1038/s42004-023-00864-y)
Supplement: Supplementary file 3 — Supplementary Data 1 [file 42004_2023_864_MOESM3_ESM.pdf]

**Supplementary Information**

# Development of pathway-oriented screening to identify compounds to control 2-methylglyoxal metabolism in tumor cells

Kouichi Yanagi, Toru Komatsu, Yuuta Fujikawa, Hirotatsu Kojima, Takayoshi Okabe, Tetsuo Nagano, Tasuku Ueno, Kenjiro Hanaoka and Yasuteru Urano

## Spectral data for characterization of compounds

$^{1}$H-NMR spectrum of dsAMC
$^{13}$C-NMR spectrum of dsAMC
$^{1}$H-NMR spectrum of Q-dsAMC
LC-MS chromatogram of Q-dsAMC

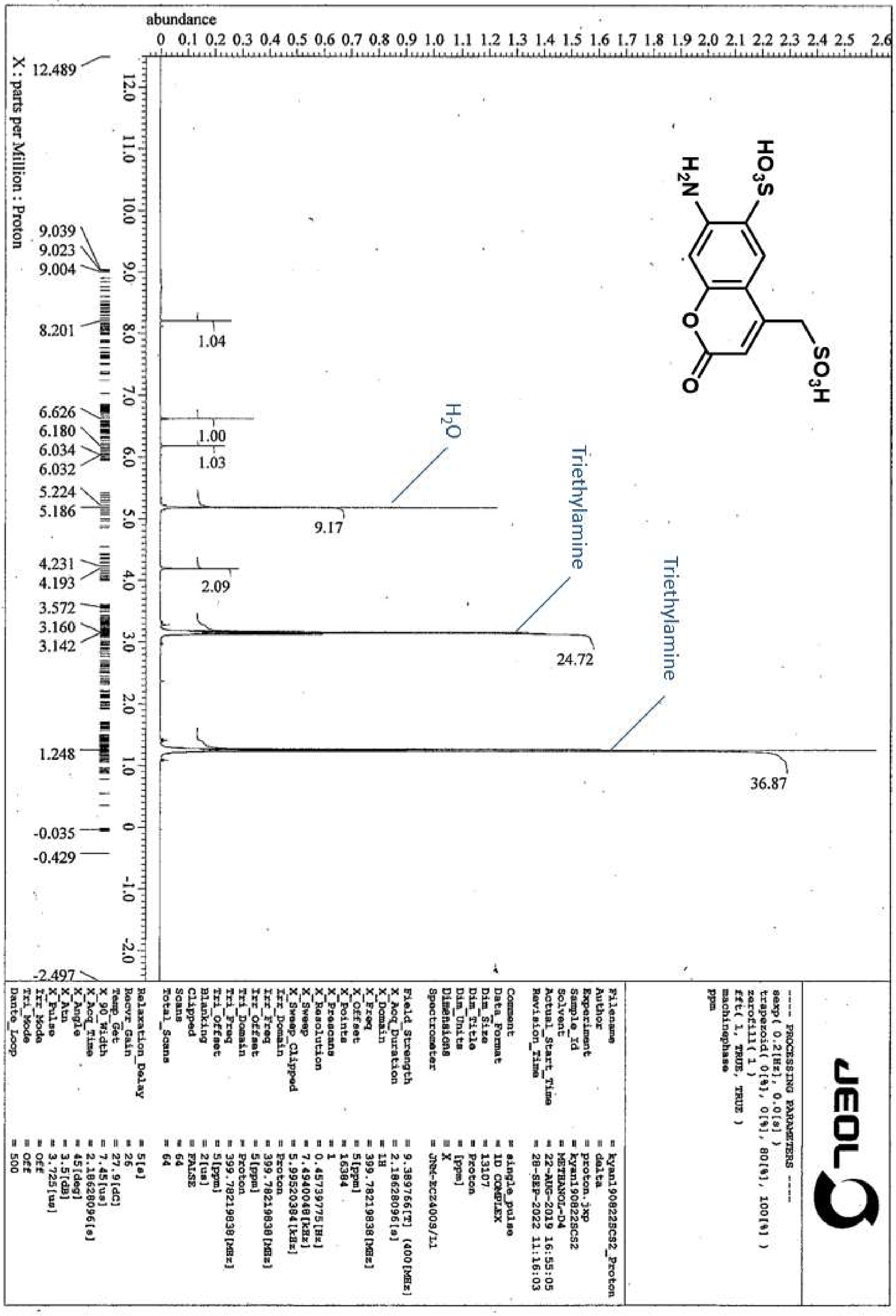


$^{1}$H-NMR spectrum of dsAMC

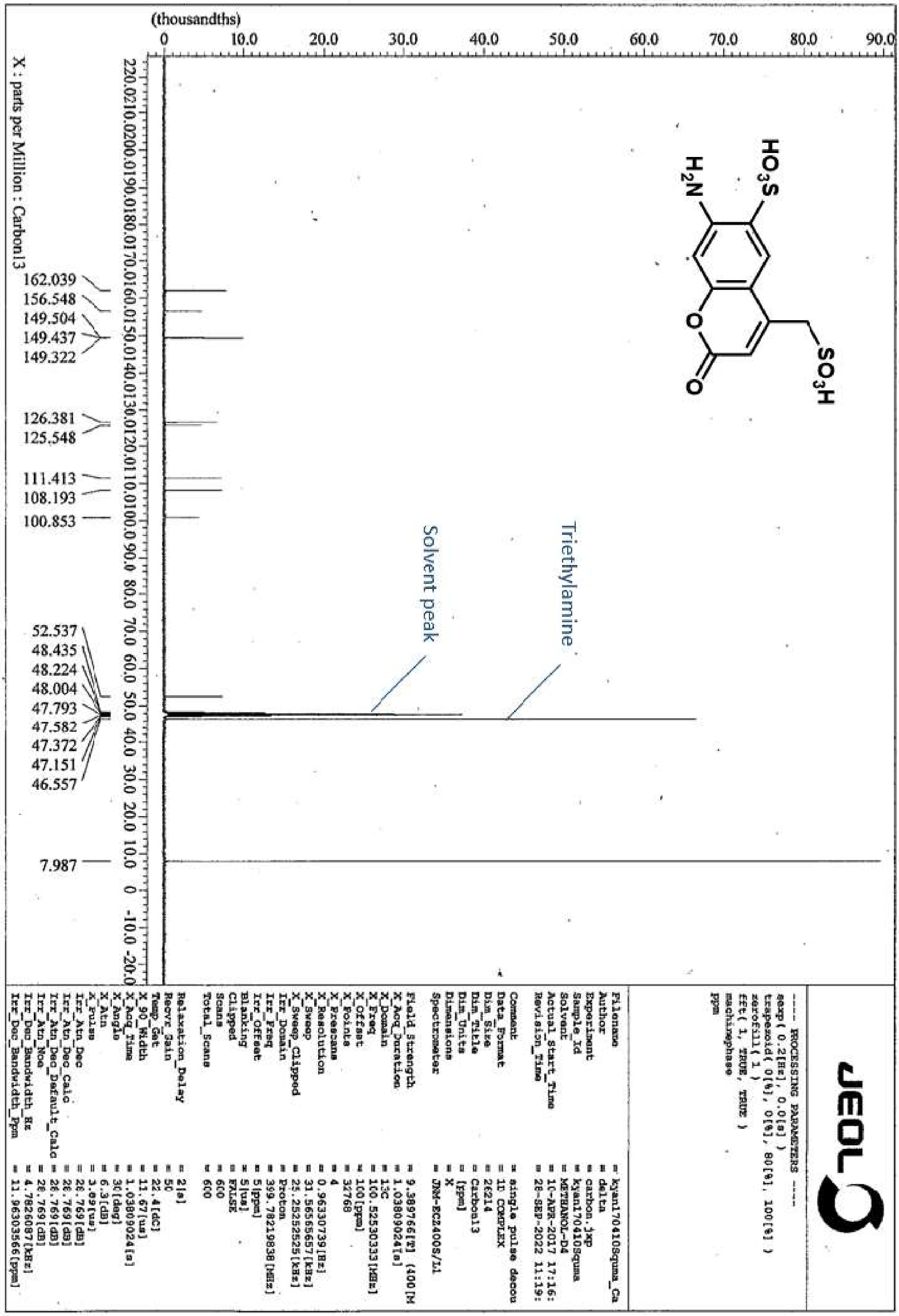


$^{13}$C-NMR spectrum of dsAMC

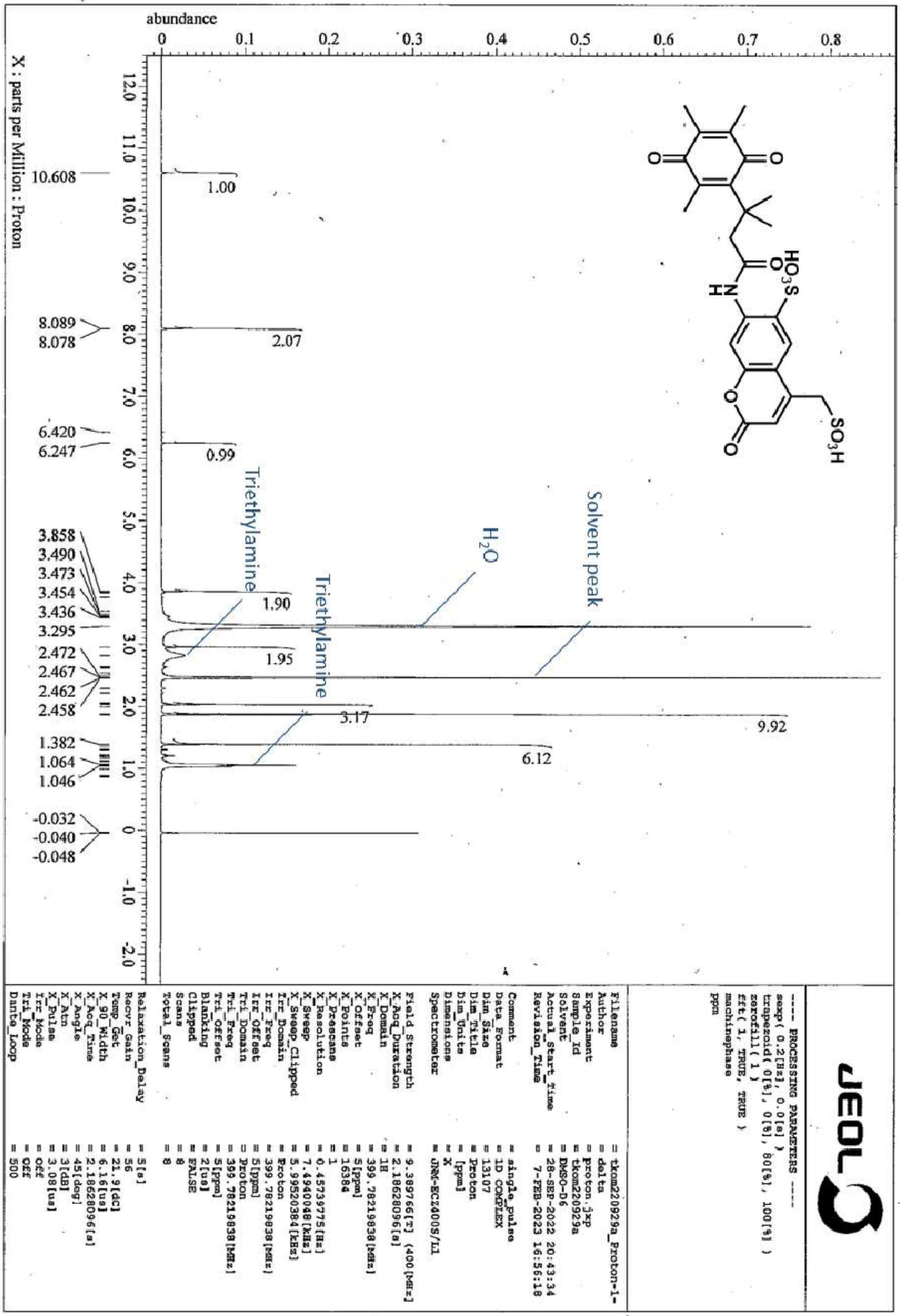


$^{1}$H NMR spectrum of Q-dsAMC

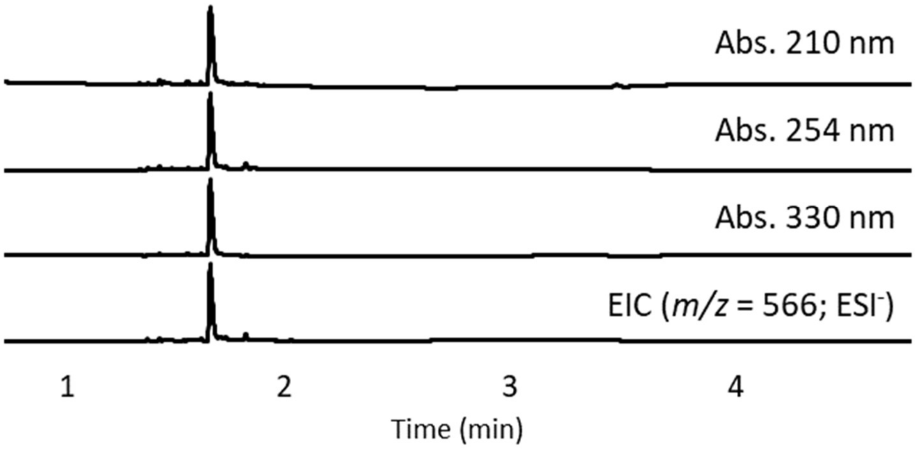


LC-MS chromatogram of Q-dsAMC

Absorbance chromatograms were monitored at 210, 254, and 330 nm, and extract ion chromatogram (EIC) was monitored at *m/z* = 566 ($ESI^-$).

Eluent: A : B= 95:5, 3.5 min, 5:95. A = 0.1% TFA aq., B = 0.1% TFA-80% AcCN-20% $H_2O$
